# Failure to diagnose hypochondroplasia by prenatal diagnosis: a case report

**DOI:** 10.1186/s12887-023-03917-2

**Published:** 2023-03-02

**Authors:** Hua Xie, Yulin Chen, Fei Xiong, Jinrong Li, Fan Yang

**Affiliations:** 1 Department of Pediatrics, West China Second University Hospital, Sichuan University, Chengdu, China; 2 Ministry of Education Key Laboratory of Birth Defects and Related Diseases of Women and Children, Sichuan University, Chengdu, China; 3 Doctorate of Health Management Program, National University of Science and Technology MISIS, Moscow, Russia

**Keywords:** Hypochondroplasia, Fibroblast growth factor receptor 3, Sanger sequencing, Case report

## Abstract

**Background:**

Hypochondroplasia (HCH) is a common nonlethal skeletal dysplasia caused by pathogenic variations in the fibroblast growth factor receptor 3 *(FGFR3*) gene, and HCH has similar clinical manifestations with achondroplasia (ACH), which can be screened during the fetal period by prenatal ultrasound testing and diagnosed by genetic testing.

**Case presentation:**

we report the special case of a patient with obvious growth retardation and rhizomelic disproportionate short stature, accompanied by other manifestations, including an enlarged head and short hands at 1 year old. However, several multiple color ultrasound exams identified shortened limbs (< 3rd percentile), an increased biparietal diameter (> 95th percentile) and a low nasal bridge in the fetal period. Due to the high incidence rate of ACH, genetic testing for the hotspot *FGFR3* gene *c.1138 g* > *A* pathogenic variations was performed immediately in the third trimester. Unfortunately, the definitive diagnosis could not be made before birth due to the negative result of hotspot gene exam. Whole exome sequencing (WES) was performed at 1 year identified *FGFR3* gene *c.1620C* > *A* variations positivity, and the patient was finally diagnosed as HCH.

**Conclusion:**

Our report extends the understanding of the limitations of prenatal genetic diagnostic testing, especially the hot spot pathogenic variations test should be not the only clinical diagnostic basis. Moreover, this case also emphasizes that further gene analysis for patients with significant conflict between the clinical manifestation and the prenatal genetic panel examination findings should be reconducted timely to spare the family from a delayed diagnosis or a misdiagnosis.

## Background

*FGFR3* gene mutation is the major pathogeny of skeletal dysplasia, including achondroplasia (ACH, OMIM: 100,800), hypochondroplasia (HCH, OMIM: 146,000), thanatophoric dysplasia types 1 (TD1) and 2 (TD2), and severe ACH with developmental delay and acanthosis nigricans (SADDAN). Major disease of this group disorders are ACH and HCH which manifest as limb shortening, short stature, and a characteristic facial configuration, leading to serious physiological and psychological adverse effects for patients [1–3]. In addition to these significant abnormal manifestations, ACH and HCH cause other characteristic clinical symptoms, including foramen magnum narrowing, ventricular enlargement, sleep apnea, upper airway stenosis, otitis media, a narrow thorax, spinal canal stenosis, spinal kyphosis, and deformities of the lower extremities [4, 5]. Although ACH and HCH have similar clinical manifestations, ACH has several additional pathologies, but the differential diagnosis depends on gene pathogenic variations detection.

Herein, we report a female proband with a short femur and a negative gene diagnosis before birth who was delay diagnosed as HCH at 1 year old. This case reveals the limitations of hot spot gene analysis as a prenatal diagnosis in some medical institution and urgent guideline of a prenatal genetic diagnostic testing strategy to avoid delayed diagnoses and misdiagnoses should be established.

## Case presentation

The patient was a girl, aged 1 year, who was admitted to our hospital due to obvious growth retardation. The girl was born to a nonconsanguineous couple, and the mother suffered from gestational diabetes during the third trimester. At 24^+3^ weeks of gestation, the color Doppler ultrasound examination of the fetus showed that one femur was shorter than that expected for healthy fetuses in the same gestational week, the ratio of femur length to plantar length was 0.81, and the proximal femur shaft-epiphysis angle was greater than 116°, which were highly suggestive of abnormal skeletal system development. Amniocentesis, high-throughput gene sequencing, prenatal screening, chromosome microarray analysis and Sanger sequencing for the *FGFR3* gene *c.1138G* > *A* pathogenic variations were performed immediately, but no obvious abnormality was found. When the genetic examination results were obtained, the fetus was already 29 gestational weeks of age. Because the findings of the prenatal diagnosis-related genetic examinations of the fetus were all negative, the family refused further whole-gene examination.

**Fig. 1:**
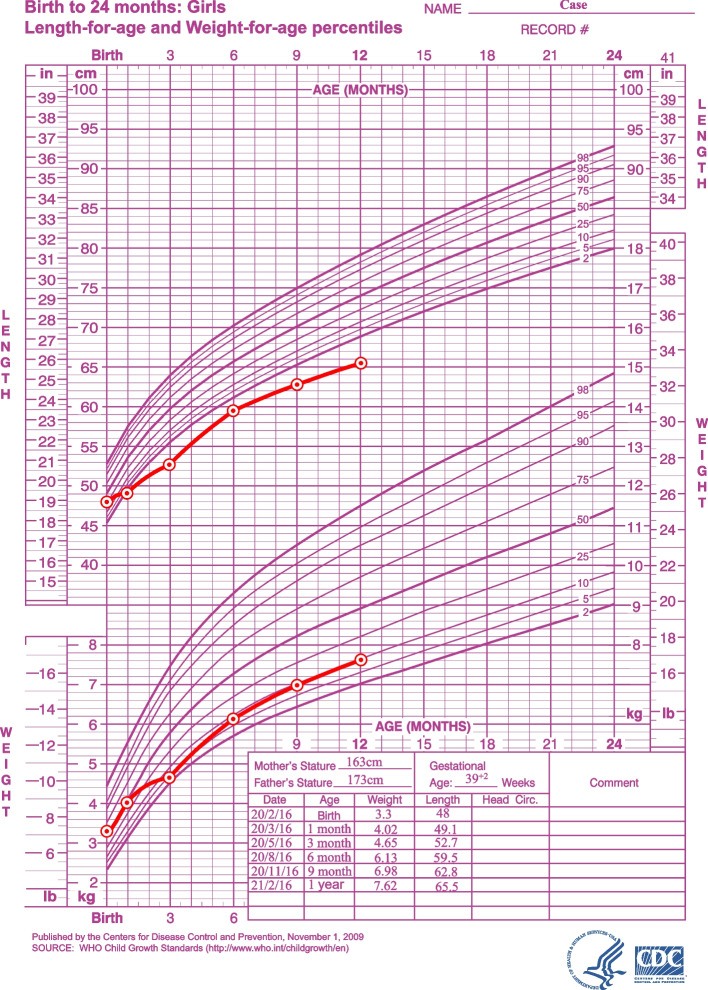
Growth Charts of the proband. The length and weight for age chart

**Fig. 2:**
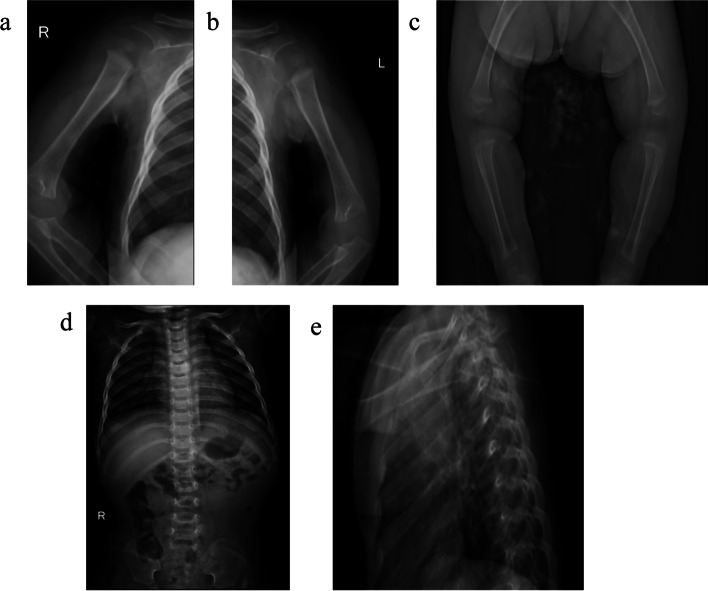
The findings on plain X-ray examination (**a**, **b**, **c**) Mild metaphyseal flaring in long bones, more evident in lower limbs, and short femoral necks. **d**, **e** spine front and lateral view. No narrowing of interpedicular distance between the first and the fifth lumbar vertebral bodies.

The proband was delivered at 39^+2^ weeks by cesarean section, with Apgar scores of 9, 10, and 10 at 1, 5, and 10 min, respectively. The mother breastfed the proband for the first ten months, and supplementary food was started at 6 months. The proband showed a poor appetite from the time of birth. The birth weight of the child was 3300 g (P55.8, Z score 0.15), and the length was 48 cm (P40.5, Z score -0.24). When she reached 6 months old, her weight was 6.13 kg (P7.5, Z score -1.44), her height was 59.5 cm (P0.3, Z score-2.76), and her cranial circumference was 45.6 cm (P97.1, Z score 1.9). At 12 months of age, her weight was 7.62 kg (P9.0, Z score-1.34), her length was 65.5 cm (< P0, Z score -3.33), and her cranial circumference was 47.5 cm (P97.1, Z score 1.9) (Fig. 1). The ratio of crown-rump length to height was greater than the upper limit of normal (Table 1). Routine test results for d-3-hydroxybutyric acid increased to 2.66 mmol/L (reference value: 0–0.27 mmol/L), while routine blood, liver and kidney function parameters and electrolyte, blood glucose, blood lactate, blood ammonia, pyruvic acid, ceruloplasmin, insulin-like growth factor-1, insulin-like growth factor-1 binding protein 3, thyroid stimulating hormone, free thyroxine, free triiodothyronine, and parathyroid hormone levels were all normal. The findings on ordinary X-ray examination showed that the bone mineral density was decreased in the tubular bones and spinal column, both humeri were thick and short, and the metaphyses of both humeri had a large width and were irregular with a cup-shaped deformity (Fig. 2). Her brain cranial magnetic resonance imaging (MRI) findings were normal. The patient was able to raise her head at 5 months old, sit with help at 8 months of age, and climb at 12 months. The Gesell Developmental Schedules test results showed that her motor development was mildly delayed due to malnutrition.

**Table 1:** The ratio of crown-rump length and height

the moon's age	Birth	1	3	6	9	12
crown-rump length(cm)	33	34.2	36.4	41.2	43.4	45.6
height(cm)	48	49.1	52.7	59.5	62.8	65.5
The ratio of crown-rump length and height	0.688	0.697	0.691	0.692	0.691	0.697

**Fig. 3:**
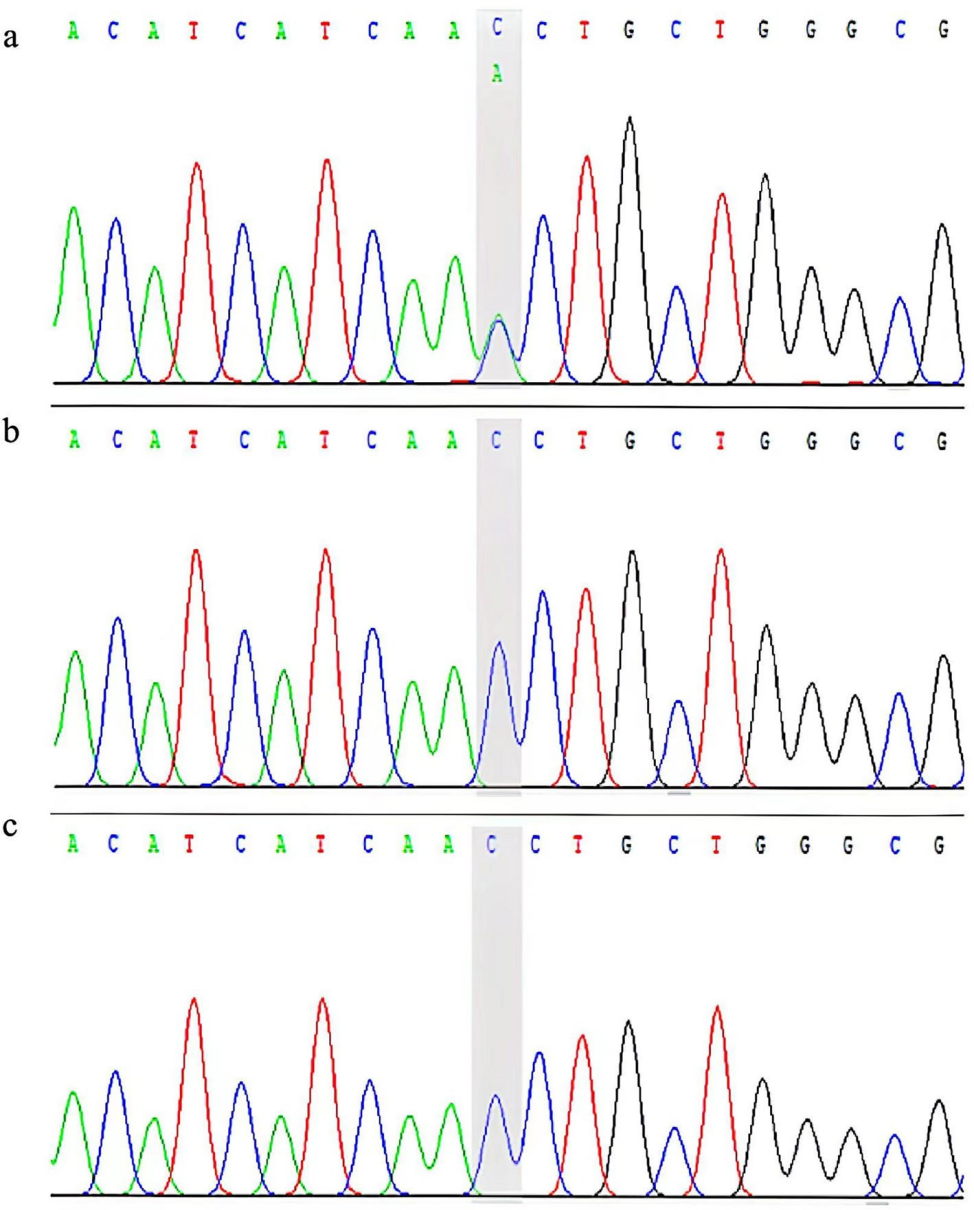
Results of the *FGFR3* analysis in the patient and her parents (**a**) A novel variant, *c.1620C* > *A*, was detected in exon 12 of the proband, which leading to an amino acid change, p. Asn540Lys. **b** No variant in *FGFR3* was detected in the mother of the child. **c** No variant in *FGFR3* was detected in the father of the child.

Fetal DNA was extracted from cells in amniotic fluid, and panel Sanger sequencing results for the *FGFR3* gene *c.1138G* > *A* pathogenic variations were negative. DNA was extracted from peripheral blood samples, and WES was performed when the proband was 1 year old. Sanger sequencing was used to verify the pathogenic variations in the proband, and the variant was classified following the 2015 guidelines of the American College of Medical Genetics and Genomics (ACMG). The *c.1620C* > *A* pathogenic variations was detected in exon 12 of the proband, which is a pathogenicity variant leading to an amino acid change, p.Asn540Lys. No relevant variants were identified in the parents, whose genotypes were normal (Fig. 3). Testing for the variant in the proband and her parents was performed by Sanger sequencing, and the variant met the criteria to be identified as a pathogenic variant, PS1 + PS2 + PS3-Supporting + PS4 + PM2-Supporting + PP3, according to the 2015 ACMG guidelines. Multiple color ultrasound examinations during the fetal period found short limbs and an abnormal diaphyseal-epiphyseal angle of the proximal femur, but the results of Sanger sequencing performed for the *FGFR3* gene *c.1138G* > *A* pathogenic variations were negative. The proband presented with asymmetrical short stature combined with an enlarged head. We performed whole-exon gene sequencing of the proband’s DNA, which was positive for the *FGFR3* gene *c.1620C* > *A* pathogenic variations, and the proband was finally diagnosed with HCH. The proband received rehabilitation training because of her delayed motor development.

## Discussion and conclusion

Achondroplasia (major pathogenic variations: *c.1138G*>*A/C*) and hypochondroplasia (major pathogenic variations: *c.1620C*>*A/G*) are both bone dysplastic diseases caused by *FGFR3* gene mutations with different hot spots pathogenic variations [1]. Due to the high disability rate and heavy burden on economy, early and timely diagnosis is very important, especially before birth [6]. Fetuses with a family history of *FGFR3* gene pathogenic variations should undergo routine prenatal genetic testing by chorionic villus sampling at 9~13 weeks of gestation or amniocentesis at 17~22 weeks of gestation based on the findings of known *FGFR3 *gene pathogenic variants in their parents [7, 8]. However, for fetuses without a family history, prenatal genetic testing should be conducted if abnormal prenatal ultrasound examination findings are identified in the third trimester of pregnancy, including short fetal limbs (below the third percentile for healthy fetuses in the same gestational week), an increased biparietal diameter (above the 95th percentile for healthy fetuses in the same gestational week) and a low nasal bridge. However, the clinical manifestations of ACH and HCH are very similar; it is difficult to make a differential diagnosis on the basis of only clinical manifestations or prenatal ultrasound exam findings, especially for intrauterine fetuses, and the final diagnosis of the disease depends on gene analysis.

The color Doppler ultrasound exam findings of our patient showed shorter femur, abnormal ratio of femur length/plantar length, larger angle of e proximal femur shaft and the metaphysis which were highly suggestive of abnormal skeletal system development; On the contrary, genetic testing for the hotspot *FGFR3* gene *c.1138 g* > *A* pathogenic variations was negative which provided no guidance for pregnancy. The conflict persist and finally be solved by whole-exome sequencing analysis for our patient at 1 year old revealed that the *FGFR3* gene *c.1620c* > *A* pathogenic variations was positive, which led to our diagnosis of HCH.

Prenatal diagnosis includes genetic examination (chromosome, gene chip, gene hot spot pathogenic variations, whole exon sequencing), imaging examination, serum markers and so on. Prenatal ultrasound examination is an important method for screening for bone dysplasia. including HCH during pregnancy, but the final diagnosis depends on genetic exam [9–11].

Unfortunately, time-consuming, incomplete clinical phenotypes (eg: special facial features) by prenatal ultrasound and tough gene polymorphism analysis make titanic difficult to obtain correct and timely exome sequencing result which can provide meaningful pregnancy guidance before birth. Without prenatal genetic diagnostic guidelines, the majority of doctors prefer to perform the hotspot pathogenic variations associated with major diseases with high incidence and mortality rates instead of exome sequencing in order to provide rapid evidence for intervention. But the choice of hotspot pathogenic variations detection are easy to miss some rare gene mutations, leading to missed diagnosis.

In conclusion, gene hotspot pathogenic variations analysis with timeliness may cause a huge risk of misdiagnosis. The limitations of gene detection should be recognized, while the results of gene hotspot pathogenic variations analysis are inconsistent with the clinical manifestations of bone dysplasia, including the prenatal ultrasound exam findings in the third trimester of pregnancy, expanded genetic testing should be conducted immediately to provide more timely and accurate guidance during pregnancy.

## Data Availability

The Datasets used and/or analyzed during the current study are available from the corresponding author upon reasonable request.

## Abbreviations

HCH: Hypochondroplasia
*FGFR3*: Fibroblast growth factor receptor 3
ACH: Achondroplasia
WES: Whole exome sequencing
OMIM: Online Mendelian Inheritance in Man
MRI: Brain magnetic resonance imaging
ACMG: American College of Medical Genetics and Genomics
